# Needs Must When—

**Published:** 1920-09-11

**Authors:** 


### THE EDITOR'S LETTER-BOX.

**Correspondence on all subjects is invited, but we cannot in any way be responsible for the opinions expressed by our correspondents, who must give their name and address as a guarantee of good faith, but not necessarily for publication. Correspondents are reminded that brevity of style and conciseness of statement greatly facilitate early insertion.**

### "Needs Must When ——."

*To the Editor of* THE HOSPITAL.

SIR,—I thank you for permitting a brief reply. A journalist is necessarily bi-sexual, and as man and woman I protest against the word "impossible" and its fatal consequences.

1. "General Practitioner" has been told by individual women in all or most classes that continence is to them impossible. What could he advise a young lady? "Marry if you can." But a considerable number cannot, even if they resort to the boldest bidding—which indeed may defeat its end. The impossible must then be achieved; its alternative is unthinkable.

2. Our English gold is still stamped with the image of a young man vanquishing a dragon—not understood to be a carted beast with cut claws and teeth extracted. Dragons take some killing; every boy knows that that is where the fun comes in (let us thank Dr. Grenfell for his application of that word). But not every lad is tuned to hero-pitch. Tell the merely average one that victory is for many impossible; will he not lean back on that comfortable word? Try telling him in the words of a man who knew the human heart pretty well that no temptation has befallen him but such as is common to man, and that he will not be tempted above that which he is able to resist. Perhaps he will perceive that it is a finer thing to play St. George's part than that of the dragon that devours maidens.

3. "General Practitioner" says that "the gratification of sexual impulse is not an anti-social deed." But that is exactly what it is, outside marriage which builds one of the cells that compose the State. If States have as yet not legislated, is it not because (*a*) preventive medicine is in its earliest infancy, (*b*) because the value of the individual citizen has only just been recognised, and perhaps (*c*) because the idea of woman as a chattel dies hard? Even so I would ask "General Practitioner's" attention to laws in the Mosaic code, which we know to have been founded on older legislation, against the manufacture of the prostitute.

4. I do not "believe" but know for a fact what I have said about schoolgirls in certain cities. The saddest feature in this case is that not poverty is the cause, but simply the desire for pocket-money, clothes, chocolates. Unfortunately the fear alike of prosecution and of disease just now give special chances to the child who contrives to look older than she is, or the girl who looks younger.

Lastly, we check the irresistible impulse to take our property by penal servitude, the overwhelming desire to get rid of inconvenient people by the gallows and the lunatic asylum. Are we always going to allow the man—or the woman—whose sex impulses are strong to rob others of all that makes life sweet, or slowly to murder them with unspeakable tortures? While we were blind—but now we say "we see."—Faithfully yours,

A SOCIAL WORKER.

